# Editorial Note: Digital transformation and supply chain efficiency improvement: An empirical study from a-share listed companies in China

**DOI:** 10.1371/journal.pone.0357932

**Published:** 2026-09-10

**Authors:** The PLOS One Editors

The *PLOS One* Editors issue this Editorial Note to inform readers that works cited in this article [1] as References 43 and 51 were retracted before [1] was published. PLOS considers that these references are not crucial in supporting the research or conclusions reported in [1].
